# A Framework for Applying Point Clouds Grabbed by Multi-Beam LIDAR in Perceiving the Driving Environment

**DOI:** 10.3390/s150921931

**Published:** 2015-08-31

**Authors:** Jian Liu, Huawei Liang, Zhiling Wang, Xiangcheng Chen

**Affiliations:** 1 Department of Automation, University of Science and Technology of China, Hefei 230026, China; E-Mail: chenxgcg@ustc.edu; 2 Institute of Applied Technology , Hefei Institutes of Physical Science, Chinese Academy of Sciences, Hefei 230026, China; E-Mails: hwliang@iim.ac.cn (H.L.); zlwang@hfcas.ac.cn (Z.W.)

**Keywords:** dynamic obstacle modeling, multi-beam LIDAR, multi-feature ground segmentation, road curb modeling

## Abstract

The quick and accurate understanding of the ambient environment, which is composed of road curbs, vehicles, pedestrians, *etc.*, is critical for developing intelligent vehicles. The road elements included in this work are road curbs and dynamic road obstacles that directly affect the drivable area. A framework for the online modeling of the driving environment using a multi-beam LIDAR, *i.e.*, a Velodyne HDL-64E LIDAR, which describes the 3D environment in the form of a point cloud, is reported in this article. First, ground segmentation is performed via multi-feature extraction of the raw data grabbed by the Velodyne LIDAR to satisfy the requirement of online environment modeling. Curbs and dynamic road obstacles are detected and tracked in different manners. Curves are fitted for curb points, and points are clustered into bundles whose form and kinematics parameters are calculated. The Kalman filter is used to track dynamic obstacles, whereas the snake model is employed for curbs. Results indicate that the proposed framework is robust under various environments and satisfies the requirements for online processing.

## 1. Introduction

Environment perception is a key research area as a source of information flow in developing unmanned ground vehicles (UGVs). Without an accurate and quick understanding of the driving environment, a vehicle is unable to make the right decisions when it moves at a high speed. Road curbs and dynamic road obstacles are the most important road elements in the driving environment. Road curbs indicate the road area for a vehicle, whereas road dynamic obstacles specify the areas to avoid.

To obtain quick and accurate understanding of the driving environment, various sensors such as cameras, stereo vision, infrared cameras, and 2D sequential lasers are employed to perceive the environment. A common problem in using these sensors is their limited description of the environment. Image processing has been studied intensively and applied extensively in modeling the driving environment to detect lanes, road boundaries, traffic lights, *etc.* [1,2,3,4,5,6,7,8]. However, unlike that of other sensors, the information provided by a camera is restricted to a certain view direction that covers a narrow field of view (FOV); moreover, distance information is lacking. Cameras are also susceptible to changing light conditions. To address the lack of distance information provided by ordinary cameras, stereo cameras have been developed and used in autonomous driving to address the lack of distance information in ordinary cameras [9,10,11]. However, stereo cameras have a narrower FOV than ordinary cameras. To overcome this light-limited disadvantage, studies on the application of infrared cameras have gained significant attention because of the capability of these cameras to detect obstacles and their insensitivity to illumination [12,13,14,15,16,17]. However, the high price and low resolution of infrared cameras limit their applications. Meanwhile, vehicles are typically equipped with a 2D sequential laser to detect obstacles in a certain direction under any weather condition [18,19,20,21,22]. However, the sparse information offered by a 2D sequential laser is insufficient for a vehicle to make its own driving decision.

To extend 2D and 2.5D to 3D, a multi-beam LIDAR is employed to replace the 2D sequential laser that can only provide points in a fixed pitch angle. Although considerable research has been conducted on 2D and 2.5D perception, only a few researchers have addressed problems in 3D perception thus far. Pieces of 2D and 2.5D information are typically represented in the form of an image, whereas a point cloud is adopted in 3D perception. The point cloud was first used in remote sensing [23,24,25,26] to model terrain. However, the method developed for remote sensing is unsuitable for UGVs because of two reasons. First, the density of the point cloud used in remote sensing is different from that of the point cloud used in UGVs. A data set is considered dense if the connectivity of scanned surfaces can be captured with the connectivity of non-empty cells (*i.e.*, cells with at least one data point), whereas empty cells exists in the sparse point cloud. The point cloud used in remote sensing is the dense point cloud, whereas what employed in UGVs is the sparse point cloud; thus, data are stored and processed differently [27]. Second, real-time requirements for remote sensing are different and significantly lower from those for UGVs. One of the most time-consuming aspects in remote sensing is determining the closest point in a point cloud because the stored points are unorganized. To overcome this problem, point structure should be considered for different types of sensors. The sensor adopted in this study is the Velodyne HDL-64E LIDAR, which is a new type of 64-beam LIDAR that is extensively used in UGVs. With its 360° horizontal FOV by 26.8° vertical FOV, 5–15 Hz user-selectable frame rate, and over 1.3 million points per second output rate, the Velodyne HDL-64E LIDAR can model the 3D environment in a point cloud. Various studies on the Velodyne HDL-64E LIDAR have been presented in literature. Some concepts in image processing, e.g., model matching, have been integrated into LIDAR data processing. Pascosl *et al.* [28] used the superquadrics fitting method to segment and model an obstacle simultaneously, whereas a plane model was applied in [29] to fit the drivable area. Range scan likelihood models were studied in [30] by directly considering the range parameter. However, the results of our experiment determined that the points obtained from the LIDAR data of a certain object varied with the relative position of the vehicle to the detected obstacle, particularly when the obstacle was far from the vehicle. In this case, only a few points were projected onto the obstacle, which caused the match to fail. Thus, the present study focuses on extracting local features from raw data using multi-line LIDAR, in which points belonging to the same obstacle are grouped together after the points that are projected onto the ground are removed. In contrast to sensors, e.g., Ibeo, which allow easy ground filtering by collecting four parallel horizontal scan lines and marking the readings that likely come from the ground [15], the data from the Velodyne LIDAR contains points projected onto the road surface. Ground segmentation methods are categorized based on the organization of a point cloud or the utilization of the information contained in a packet. In the first category, the vicinity information in raw data is not considered and only the positions of the points are obtained. Azim *et al.* [31] determined that a pratical means to identify a dynamic obstacle was to detect the change in occupancy in an octree. Meanwhile, a 1D Gaussian process (GP) regression with a non-stationary covariance function was used to distinguish the ground points or obstacles in each segment of a polar coordinate system in [32]. Excellent results based on the vicinity information in the packet obtained from the Velodyne LIDAR were observed after the 2004 and 2005 Grand Challenges and the 2007 Urban Challenge held by the Defense Advanced Research Projects Agency to boost the development of UGVs. Von Hundelshausen *et al.* [33] proposed an obstacle detection method based on the different values of points located within the same grid cell produced by a single beam. This method was also applied in [34]. The number of points projected onto the same grid and the height difference in the same grid were considered in [35]. Height difference was also employed in [36,37,38], with the addition of the range comparison returned by two adjacent beams presented in [21]. Moosmann *et al.* [36] projected a point cloud onto a cylinder whose axis was the rotational axis of the scanner; the local convexity criterion was applied to segment the ground. In our experiment, various features were tested and a conclusion was presented, that is, multi-features with a loose threshold should be considered to address challenges in various environments.

After an obstacle is detected, the dynamic obstacles and road curbs are tracked. Tracking multiple dynamic objects is a complex problem that is generally divided into two parts: data filtering and data association. Filtering is the sequential estimation of the state of a dynamic object. This process is typically performed using Bayesian filters and requires a specific motion model to predict the positions of tracked models in an environment. After predicting the positions of existing tracks, data association is performed to assign observations to existing tracks. Although the framework is the same as the one previously mentioned, details vary. A bounding box was employed to classify the characteristics of the obstacle, and the global nearest neighbor was applied in data association to predict obstacles in [31]. The Junior [37] tracked an obstacle by identifying the area where changes occurred; a set of particles were then initialized as possible object hypotheses to implement rectangular objects with various dimensions at slightly different velocities and locations to track the dynamic obstacle. Another proposed approach was grouping the classified obstacle range returns into local line features that are tracked across consecutive scans using a multiple-hypothesis Kalman filter [39]. In road curb tracking, two typical Bayesian methods were combined in [21], namely, the interacting multiple model-probabilistic data association filter approach. Another work [40] focused on the particle filter.

A framework for detecting and tracking road curbs and dynamic obstacles is presented in this study. First, ground segmentation is performed with dynamic obstacles clustered simultaneously by combining multi-features from both the point cloud and the obstacle grid map generated from the point cloud. This process was tested robustly in various urban and rural environments. A general approach to detect obstacles is ground segmentation followed by obstacle clustering. However, a novel method is presented in the current work wherein obstacles are clustered during ground segmentation, which reduces time consumption. In addition, the features extracted from the Velodyne LIDAR raw data are studied comprehensively in this section. To our knowledge, this work is the first attempt to conduct such a study. Second, geometric parameters are obtained separately for dynamic obstacles and road curbs. Local information is applied to dynamic obstacle detection using the Karhunen–Loeve transformation, whereas global information is applied to road curb detection using distance transformation. Third, the processes involved in the tracking procedures for dynamic obstacles and road curbs are completely different. A Kalman filter is employed for dynamic obstacles, whereas road curbs are fitted using the snake model. The Kalman filter has been proven to be a minimum-variance state estimator for linear dynamic systems with Gaussian noise and the best linear estimator for non-Gaussian noise [41], which is suitable for this work. Moreover, the improved real-time performance of the proposed approach compared with other Bayesian filters, e.g., particle filter, makes it applicable to high-speed unmanned vehicles. The snake model is selected for its capability to combine local curvature information with overall continuous information. To our knowledge, tracking and detecting dynamic obstacles and road curbs are performed separately in previous works. By contrast, these two processes are combined in the current work. In the proposed framework, previous information can be applied to detect obstacles.

The succeeding portions of this paper are organized as follows: The dynamic obstacle and road curb detection process are described in Section 2. The process of tracking road curbs and dynamic obstacles is discussed in Section 3. The experimental procedures are presented in Section 4.

## 2. Detecting Road Curbs and Dynamic Obstacles

### 2.1. Ground Segmentation

**Figure 1:**
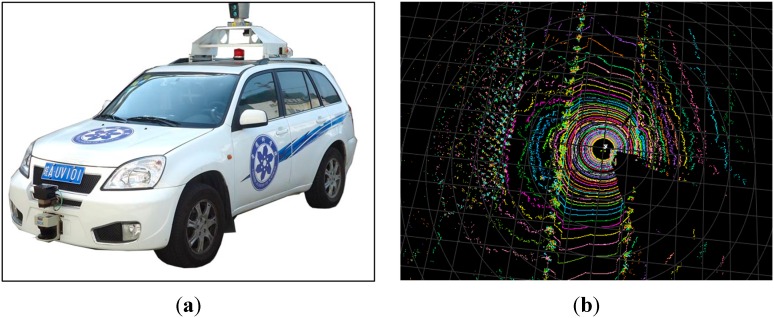
(**a**) The test car; (**b**) A point cloud frame.

As described earlier, the Velodyne LIDAR provides a comprehensive description of the ambient environment. It was employed in our experiments as follows. The Velodyne LIDAR was mounted on the test car, called *Intelligent Pioneer*, and the direction of the vehicle was marked as the starting direction from which 64 points would be sampled from the 64 lasers every 0.2°. Thus, a point cloud frame that consisted of 1800 × 64 3D points that described the all-around car environment would be obtained after one spin of the Velodyne LIDAR. The coordinates of the points were translated from the polar coordinate system into the Euclidean coordinates, wherein the up direction was set as the *z* axis and the forward direction was set as the *y* axis. To utilize the information in the raw data sent by the Velodyne LIDAR, the points were stored in an 1800 × 64 2D matrix called *Clouds*. In this matrix, the column represents a circle of points generated by one laser in one spin, whereas the row represents 64 points generated by 64 lasers in one rotating position. The 1800 points generated by one laser in a single spin that was projected onto the flat horizontal ground would form a circle. By contrast, the 64 points generated by the 64 lasers in a fixed rotating position would form a straight line. The tested car and the point cloud frame obtained using the Velodyne LIDAR are shown in Figure 1.

Based on the preceding analysis, road curbs or dynamic obstacles can be regarded as consisting of points that are not in the position they should have been if projected onto a flat horizontal plane. If the points are projected onto such a plane, the points will form concentric circles, and changing the positions of the points of the obstacles will affect local geometric characteristics and prevent concentric circles from forming. That is, detecting obstacle points is modeled as removing points projected onto a road surface, the local geometry of which is similar to those projected onto a flat horizontal plane. However, a road surface is not an entirely horizontal plane and the effects of various lasers are different because of varying scan ranges; hence, segmenting ground points by using only a single characteristic is difficult. When only a single characteristic is applied, a strict threshold causes leak detection of imperceptible obstacles, whereas a loose threshold results in false detection, particularly in field environments where road conditions are complex. The principle behind our work involves applying various features and intensively testing each feature using a relatively loose threshold. Given that the scan frequency is 10 Hz and the local features are only affected by neighboring points, which are generated nearly simultaneously, the ego-motion of the vehicle has minimal influence on the local feature. The features applied in this work are described in the following sections.

#### 2.1.1. Change in Radius between Neighboring Points in One Spin

The most significant feature for a circle formed by one laser in one spin is the nearly similar distances between the LIDAR and the points. However, the distance will change significantly when the laser beam is blocked during rotation. Thus, the ratio between the radii of neighboring points in one circle can be designated as a feature. If the ratio is within $\left[1-{\text{δ}}_{1},1+{\text{δ}}_{1}\right]$, then the point is designated as a road surface point and is removed.

#### 2.1.2. Detecting Broken Lines

This method extracts line segments from the raw data obtained from the sensor in the polar coordinates. The line segments are classified into road and obstacle segments. A detailed description of this method is provided in [20]. This feature is applied to detect only straight lines alone as a supplement of the points filtered by other features. The points between two broken lines are fitted with a line model. The points will be designated as non-ground if the line model fits well. This feature is particularly applicable to boundary points with a straight model, e.g., straight road curbs, vehicle edges, *etc.* However, it will fail when applied to curved boundaries.

#### 2.1.3. Tangential Angle

**Figure 2:**
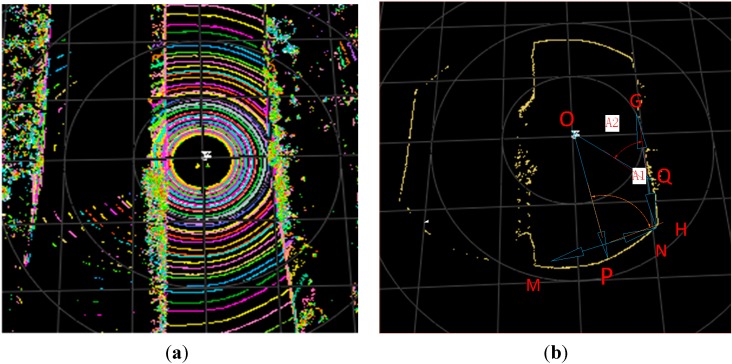
(**a**) A sample point cloud; (**b**) the points formed by one laser in one spin.

As mentioned earlier, a circle will form if the points generated by the same laser in one spin are projected onto a flat horizontal plane. However, this process will not occur if an obstacle exists. This situation is illustrated in Figure 2.

The figure depicts a driving environment on an urban road whose boundaries are formed by a parterre. Figure 2b shows a portion of Figure 2a. Hence, Figure 2b is formed by one laser in one spin, whereas Figure 2a is formed by a total of 64 lasers in one spin.

As shown in Figure 2b, situation varies according to projection position. Based on this figure, if a point , e.g., point *P*, is projected onto a road surface, then the tangential angle formed by its radial direction, *i.e.*, $\vec{OP}$, and its tangential direction, which is expressed by two of its symmetrical neighboring column points in *Clouds*, *i.e.*, $\vec{MN}$, is nearly perpendicular*.* Otherwise, if the point, e.g., point *Q*, is projected onto an obstacle, then its radial direction, *i.e.*, $\vec{OQ}$, and its tangential direction, *i.e.*, $\vec{GH}$, will form an acute angle. Thus, ground segmentation can be described as follows: if the absolute value of the cosine of the tangential angle is less than the threshold ${\text{δ}}_{2}$ , then it is regarded as a road surface point and is removed.

This feature is particularly applicable to obstacles that are far from the vehicle because the farther a point is, the larger the value of the tangential angle formed by the obstacle.

#### 2.1.4. Local Height Difference

Assuming that the road is flat; the height difference in a local area will then be small. Nevertheless; an obstacle causes a sudden change in height. The point cloud is projected onto a 512 × 512 grid map; wherein each pixel covers a range of 20 cm × 20 cm. Hence; a range of approximately 100 m × 100 m of the environment will be covered. The maximum height difference will be calculated in each pixel. The pixel will be marked as an obstacle area if the height difference exceeds the threshold ${\text{δ}}_{3}$.

#### 2.1.5. Gradient in the Radial Direction with a Dynamic Threshold

As mentioned earlier, the 64 points generated by 64 laser beams in one direction will form a straight line when they are projected onto a flat plane. However, the line will be broken if it is blocked by an obstacle. Thus the gradient in the radial direction will be changed. The dynamic threshold of the change in gradient is obtained from the inner points in this direction by tripling the variance. A detailed description of this work is provided in [42].

#### 2.1.6. Determining the Threshold of the Features

**Figure 3:**
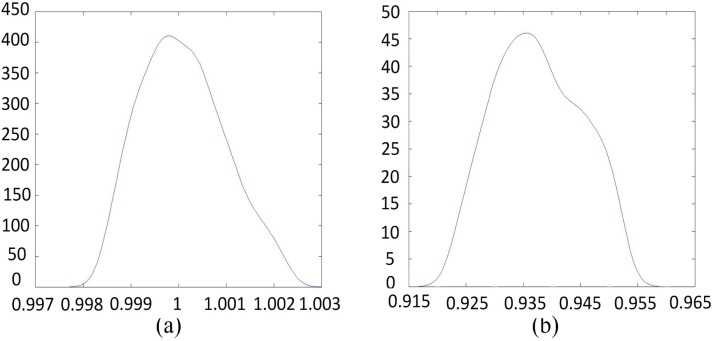
Distributions for features 1 (**a**) and 3 (**b**) of points projected onto the road surface.

A total of 17 features are tested in our experiment. The features mentioned from Section 2.1.1, Section 2.1.2, Section 2.1.3, Section 2.1.4 and Section 2.1.5 are proven to be effective and robust, whereas other features cause leak detection of the obstacles. For example, the height difference between neighboring points in one spin from a laser, which is a feature adopted in many works, will remove distant road curb points because height difference changes gradually when points are projected. To provide an analogy, if the height of a road curb is 10 cm and up to 10 points are projected onto it from one laser in one spin, which is possible in an actual situation, then the average height difference is 1 cm. Thus, the point will be removed. To determine the threshold for the first and third features, an experiment was conducted to obtain the statistics for the distribution of the feature values of the points projected onto the ground. The result is shown in Figure 3.

As shown in the figure, the range for the change in radius between neighboring points in one spin is [0.997, 1.003]. Thus a looser threshold of 0.05 is adopted in this work. Although the range for the third feature is [0.915, 0.965], our experiment demonstrates that a threshold with a value near 1 removes the points belonging to non-ground points near the vehicle. Therefore, a considerably looser threshold of 0.6 is adopted. The threshold for the fourth feature was set to 15 cm in [38]. However, a looser threshold of 10 cm is adopted in this work to detect unobvious obstacles. Details are shown in Table 1.

**Table 1:** Thresholds adopted in the experiment.

**δ_1_ for feature in** Section 2.1.1	0.05
**δ_2_ for feature in** Section 2.1.3	0.6
**δ_3_ for feature in** Section 2.1.4	10

### 2.2. Obstacle Clustering

**Figure 4:**
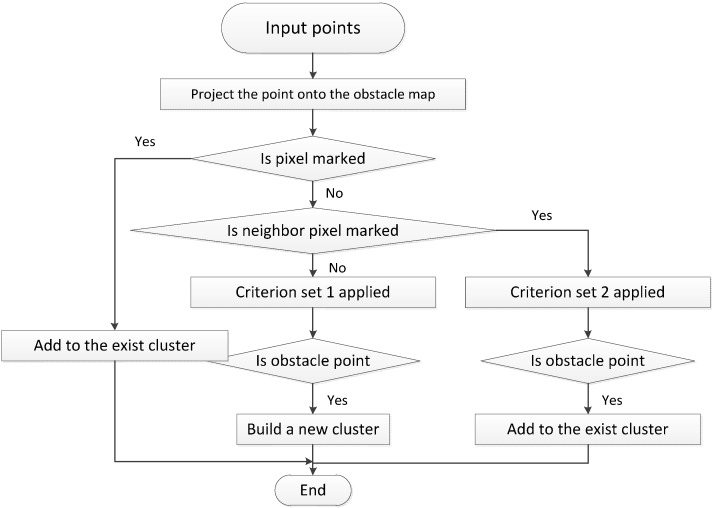
Process flow for clustering.

To our knowledge, previous works have processed road surface segmentation and obstacle clustering separately. By contrast, clustering and ground segmentation are performed simultaneously in our work. Hence, a point is assigned to an obstacle point cluster once it is detected. The proposed method offers two advantages over previous approaches. First, processing time is reduced because an extra obstacle clustering step is eliminated. Clustering is conducted during obstacle detection and the position of the test point can be obtained; thus, clustering can be performed locally instead of globally. Second, previous clustering information can be utilized in subsequent detection. This advantage is demonstrated in our experiment. Neighboring information can also be regarded as a feature to detect obstacles. For example, the points on top of a bus will be removed if all criteria are satisfied. However, if the boundary of the bus is detected after all the criteria are satisfied, then the points on top of the bus can be detected using a looser threshold because detecting the boundary of the bus suggest that neighboring points are likely to be obstacle points. If the neighbor of a point is designated as an obstacle, then the point can be identified as an obstacle with less constraint. In our experiment, if no obstacle point is detected among the neighbors of the tested point, then the combination of all the criteria mentioned in Section 2.1 except for the second criterion, called criterion set 1, should be satisfied to designate the point as an obstacle point. Otherwise, only the combination of the local height difference and tangential angle, called criterion set 2, should be satisfied. An ID is attached to each obstacle cluster during the detection process and an obstacle map is used to record the clusters that have been built based on the grid map. However, unlike that in the grid map, the information registered in an obstacle map is the ID of the cluster to which the pixel belongs to. Moreover, a list of detected clusters is maintained and updated during the process. The process flow is shown in Figure 4.

### 2.3. Calculating Obstacle Cluster Shape Characteristics

A number of clusters are obtained after the process described in Figure 4 has been performed. For each cluster ${\text{ζ}}_{k}$ that consists of points $\left\{(x_{i},y_{i},z_{i})\right\}$, with *i* = 1,2,3,…,*N*, the shape characteristic for the projection on the *x – y* plane is calculated as follows:

(1) The center position $O_{0}^{k}$ of the cluster is calculated as follows:
  (1) $$centerX_{0}^{k}=\frac{1}{N}\sum_{i=1}^{N}x_{i}$$
  (2) $$centerY_{0}^{k}=\frac{1}{N}\sum_{i=1}^{N}y_{i}$$
(2) The covariance matrix in the *x* – *y* plane is calculated as follows:
  (3) $$C_{0}^{k}=\frac{1}{N}\sum_{i=1}^{N}\left[x_{i}-centerX_{0}^{k},y_{i}-centerY_{0}^{k}\right]{\left[x_{i}-centerX_{0}^{k},y_{i}-centerY_{0}^{k}\right]}^{T}$$
(3) The eigenvalue and eigenvector of $C_{0}^{k}$ are calculated and saved in matrices *P* and *X*:
  (4) $$\left\{ \begin{array}{l} P_{0}^{k}=\left[\begin{array}{cc} \lambda_{0}^{1,k} & 0\\ 0 & \lambda_{0}^{2,k} \end{array}\right]\\ X_{0}^{k}=\left[\begin{array}{cc} {\vec{n}}_{0}^{1,k} & {\vec{n}}_{0}^{2,k} \end{array}\right]=\left[\begin{array}{cc} x_{0}^{1,k} & x_{0}^{2,k}\\ y_{0}^{1,k} & y_{0}^{2,k} \end{array}\right]\\ C_{0}^{k}X_{0}^{k}=X_{0}^{k}P_{0}^{k} \end{array}\right.$$

As proven in the Karhunen-Loeve transformation, the principal component denotes the direction in which the cluster points are irrelevant. The principal component can be obtained by transforming the rectangular axis through rotation transformation defined by the eigenvector of the covariance matrix, *i.e.*, $X_{0}^{k}$. The dynamic road obstacle are mostly vehicles, which can be represented by a cuboid in 3D or a rectangle in 2D. Thus, the forward direction of the dynamic obstacle can be represented by its irrelevant direction, *i.e.*, ${\vec{n}}_{0}^{1,k}$, in 2D.

The *x* – *y* points in ${\text{ζ}}_{0}^{k}$ are projected onto two directions defined by ${\vec{n}}_{0}^{1,k},{\vec{n}}_{0}^{2,k}$, which are perpendicular to each other. Half of the length and width can be determined by calculating the maximum distance of the projection onto the center. Hence, the minimum bounding box can be obtained in the obstacle map. Moreover, the height can be determined by calculating the maximum difference in the *z* direction. The height, length, and width of an obstacle are saved in the state variables $H_{0}^{k}$, $L_{0}^{k}$, and $W_{0}^{k}$, respectively.

### 2.4. Detecting Road Curbs

**Figure 5:**
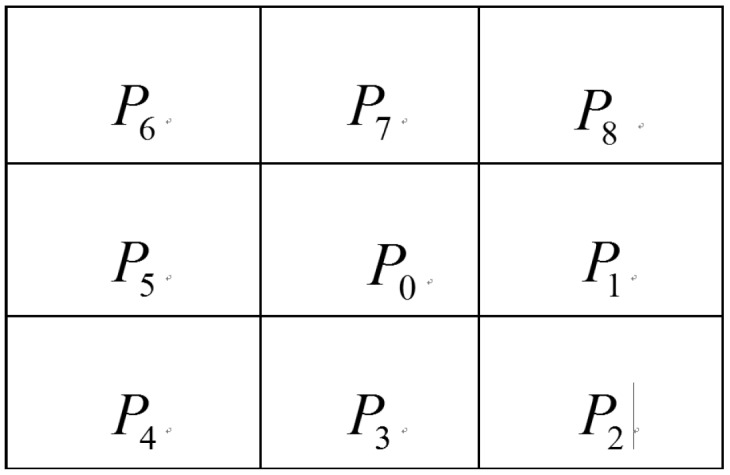
$P_{0}$ and its eight neighboring pixels.

The most significant feature of a road curb is its continuity. However, local discontinuity may affect this feature. Discontinuity may result from natural road curbs or the leak detection of a road curb because it is unobvious. To enhance the continuity information of a road curb and reduce local discontinuity, a smoothing filtering technique is employed on the grid map. Filtering is performed through the following steps:

(1) As a key step, binarization is conducted on the obstacle map wherein obstacle pixels are marked as 0, whereas other pixels are designated as 255. This step is followed by smoothing filtering, in which the possibility of a pixel marked as a road surface is designated as its intensity. The intrinsic logic of filtering is as follows: when a pixel is far from the pixel marked as an obstacle, then the possibility that it is a road surface increases. In the map, the difference between the pixel and its neighboring eight pixels should not exceed 2. To satisfy this restriction, intensity difference threshold filtering is performed from the top left corner to the bottom right corner and then from the bottom right corner to the top left corner. To illustrate this step, a pixel and its eight neighboring pixels are shown in Figure 5.

In the first iteration from the top left corner to the bottom right corner, the effect of pixel $P_{0}$ on its neighboring pixels is described as follows:

(5) $$P_{i}=\min\{P_{0}+2,P_{i}\}\;i=1\sim 4$$

In the second iteration from the bottom right corner to the top left corner, the effect of pixel $P_{0}$ on its neighboring pixels is described as follows:

(6) $$P_{i}=\min\{P_{0}+2,P_{i}\}\;i=5\sim 8$$

(2) A threshold filter is used on the map to identify the road area. The threshold is set to 20 in this work. A pixel value larger than 20 is set to 0; otherwise, it is set to 255.
(3) A neighboring road pixel search algorithm is employed to detect the road area. The algorithm starts from (256, 100), which is the position of a vehicle, to search the eight neighboring pixels. The pixel is designated as a road surface pixel only if all of its 24 neighboring pixels in a 5 × 5 grid are 0. Once a pixel is designated as a road surface pixel, it is then added to the road surface area and a search is performed on its eight neighboring pixels iteratively. A drivable area will be identified at the end of the iteration. To accelerate the process, the search is restricted to a rectangular area in the vehicle forward direction.
(4) A road curb is identified by searching the boundary of the road area, which is described in detail in our previous work [42], and stored in $C=\{P^{i},i=1\sim N\}$. Finally, a least square fit is applied to *C* to form a quadratic curve.

**Figure 6:**
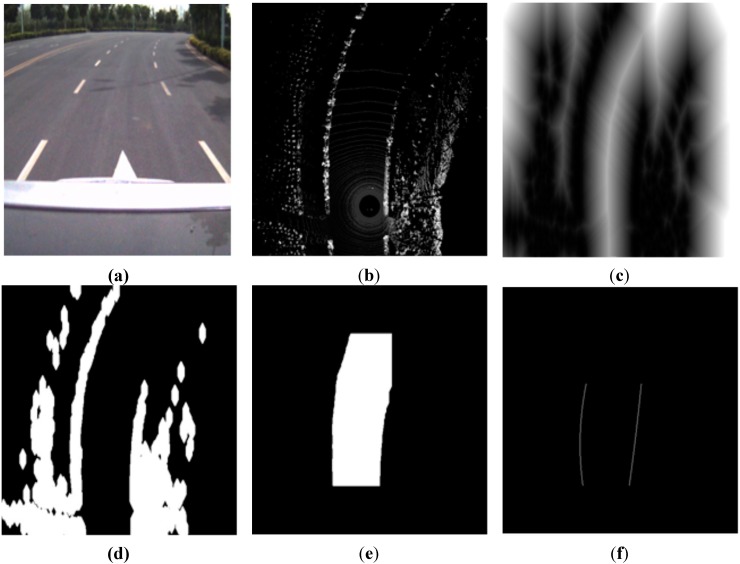
Example of road curb detection. (**a**) Original image obtained by the camera that describes the local environment; (**b**) Original grid map obtained by the Velodyne LIDAR; (**c**–**f**) Results for Steps 1–4, respectively.

An example of this process is shown in Figure 6.

## 3. Tracking Road Curbs and Dynamic Obstacles

Tracking is a complex problem that can be divided into two steps: prediction and updating the prediction. Prediction is the sequential estimation of the state. It is typically performed using Bayesian filters that require a specific motion model to predict the positions of the tracked objects in the environment. After predicting the positions of existing tracks, data association is performed to assign the observations to existing tracks. The tracking of road curbs and dynamic obstacles is addressed separately in our work. A road curb is a static obstacle in the environment; hence, the variation of its position between different frames merely depends on the motion of a vehicle. For dynamic obstacles, however, the variation in state depends on both the motion of a vehicle and the state of the dynamic obstacles. The motion information of a vehicle can be obtained from the Global Positioning System (GPS) and inertial navigation systems (INSs). The processes of tracking road curbs and dynamic obstacles are described in detail in the following sections.

### 3.1. Tracking Road Curbs

#### 3.1.1. Predicting Road Curbs

As mentioned earlier, a road curb can be considered a static obstacle and predicting a road curb merely depends on the motion states of a vehicle. The motion of a vehicle is simultaneously modeled as a transfer motion and a rotating motion. The motion of the road curb can be modeled as the relative motion in the negative direction of the vehicle, which can be obtained directly from Synchronous Position, Attitude, and Navigation (SPAN)-CPT. The SPAN technology combines two different but complementary technologies: a global navigation satellite system (GNSS) and an Inertial Navigation System (INS). The absolute accuracy of GNSS positioning and the stability of Inertial Measurement Unit (IMU) gyro and accelerometer measurements are tightly coupled to provide an exceptional 3D navigation solution that is stable and continuously available, even through periods when satellite signals are blocked. The state space equation for this static model is presented to model the road curb motion $C=\{P^{i},i=1\sim N\}$ as follows:

(7) $${\hat{P}}_{k+1}^{i}=A_{k}P_{k}^{i}+B_{k}$$

where:

C: pixel set that consists of a road curb,
$P^{i}={\left(x^{i},y^{i}\right)}^{T}$: a curb pixel,
$A_{k}=\left[\cos({\text{ϑ}}_{v,k})\sin({\text{ϑ}}_{v,k})-\sin({\text{ϑ}}_{v,k})\cos({\text{ϑ}}_{v,k})\right]$,
$B_{k}=\left[-(\Delta x_{v,k}+x_{v,k})\cos({\text{ϑ}}_{v,k})-(\Delta y_{v,k}+y_{v,k})\sin({\text{ϑ}}_{v,k})+x_{v,k}(\Delta x_{v,k}+x_{v,k})\sin({\text{ϑ}}_{v,k})-(\Delta y_{v,k}+y_{v,k})\cos({\text{ϑ}}_{v,k})+y_{v,k}\right]$,
${\text{ϑ}}_{v,k}$: rotation of the vehicle,
$x_{v,k},y_{v,k}$: *x* and *y* coordination of the vehicle in the map,
$\Delta x_{v,k},\Delta y_{v,k}$: transformation in the *x* and *y* directions.

#### 3.1.2. Updating the Prediction of a Road Curb

To increase the accuracy of the predicted road curb points, the predicted road curb should converge to the road curb in this frame by adopting the result of ground segmentation. As mentioned earlier, the most significant feature of a road curb is its continuity. However, local discontinuity occurs because of two reasons. First, discontinuity is caused by the scene, e.g., the adjacent parterre. Second, leak detection of a road curb is inevitable because road curbs are not obvious obstacles that may be concealed by dynamic obstacles on the road. Considering global continuity and local discontinuity, the snake algorithm is applied to update prediction.

The snake model was proposed by Kass [43]. A snake is an energy-minimizing spline guided by external constraint forces and influenced by image forces that pull it toward features such as lines and edges. The energy function is defined by the integration of three items, as follows:

(8) $$E_{snake}=\int E_{\mathrm{int}}(\nu (s))+E_{image}(\nu (s)))ds$$

where $E_{\mathrm{int}}(\nu (\text{s}))=(\alpha (s)|\upsilon_{s}(s){|}^{2}+\beta (s)|\nu_{ss}(s){|}^{2})/2$ represents the internal energy of the spline because of bending, and $E_{image}(\nu (s))=-\gamma (s)|\nabla I(\nu ){|}^{2}$ denotes the external energy that corresponds to the edge features of the image. Parameters α(s),β(s),γ(s) denote the weighting coefficients of elastic energy, curvature energy, and external energy, respectively; whereas ν(s)=(x(s),y(s)) represents the pixel coordinates on the spline.

To decrease the time consumed in updating the road curb, the elevation map is applied. Some noise points are detected in the grid map because only the height difference feature is applied to update prediction. This decision is based on two considerations. First, the elevation map can be obtained immediately once the LIDAR point is parsed, which can improve real-time performance. Second, the height difference in our experiment is relatively robust and only a few discrete noise points remain, which will not affect the final result because the snake model considers global continuity information. The iteration starts from the predicted road curb point obtained using Equation (7) and continues until the local minimum value of *E_snake_* is obtained. Our experiment proves that the curve converges to the road boundary when the minimum value of the *E_snake_* is obtained.

For a rapid convergence, the greedy snake [44] is adopted in our work. The vehicle dynamic information obtained by GPS and INS is accurate and a road curb is a static obstacle; hence, the local optimum value is sufficient to converge to the actual road curb in our experiment.

### 3.2. Tracking Dynamic Obstacles

The motion state of a dynamic obstacle in a frame reflects its relative position with a vehicle. Thus, its motion state depends on both the dynamic obstacle and on itself. Unlike a road curb whose motion state between frames can be obtained from the sensors in a vehicle, a motion state vector is maintained for each tracked dynamic obstacle. The track of dynamic obstacles is modeled as a linear time invariant system in our experiment, and the Kalman filter is used.

The motion state for each dynamic obstacle is composed by and given as:

$$S_{t}^{k}={\left[x_{t}^{k}y_{t}^{k}v_{t}^{x,k}v_{t}^{y,k}a_{t}^{x,k}a_{t}^{y,k}{\text{ϑ}}_{t}^{k}\alpha_{t}^{k}L_{t}^{k}W_{t}^{k}\right]}^{T}$$

where $O_{t}^{k}=(x_{t}^{k},y_{t}^{k})$ is the center position of a dynamic obstacle; $\vec{v_{t}^{k}}=(v_{t}^{x,k},v_{t}^{y,k})$ is the velocity of a dynamic obstacle; $a_{t}^{k}=(a_{t}^{x,k},a_{t}^{y,k})$ is the acceleration of a dynamic obstacle; ${\text{ϑ}}_{t}^{k}$ is the forward direction of a dynamic obstacle; $\alpha_{t}^{k}$ is the angle acceleration of a dynamic obstacle; and $L_{t}^{k},W_{t}^{k}$ are the length and width of a dynamic obstacle, respectively. To initialize $S_{t}^{k}$, once a new cluster $O_{t}^{k}=(x_{t}^{k},y_{t}^{k})$ is detected, ${\text{ϑ}}_{t}^{k}$ and $L_{t}^{k},W_{t}^{k}$ can be obtained through the process presented in Section 2.3 and the others are initialized as 0.

The dynamic obstacle motion system is modeled as follows:

(9) $$S_{t+1}^{k}=AS_{t}^{k}+w_{t}^{k}$$

(10) $$Z_{t}^{k}=S_{t}^{k}+v_{t}^{k}$$

where A = $\left[\begin{array}{cccccccccc} 1 & 0 & T & 0 & \frac{1}{2}T^{2} & 0 & 0 & 0 & 0 & 0\\ 0 & 1 & 0 & T & 0 & \frac{1}{2}T^{2} & 0 & 0 & 0 & 0\\ 0 & 0 & 1 & 0 & T & 0 & 0 & 0 & 0 & 0\\ 0 & 0 & 0 & 1 & 0 & T & 0 & 0 & 0 & 0\\ 0 & 0 & 0 & 0 & 1 & 0 & 0 & 0 & 0 & 0\\ 0 & 0 & 0 & 0 & 0 & 1 & 0 & 0 & 0 & 0\\ 0 & 0 & 0 & 0 & 0 & 0 & 1 & T & 0 & 0\\ 0 & 0 & 0 & 0 & 0 & 0 & 0 & 1 & 0 & 0\\ 0 & 0 & 0 & 0 & 0 & 0 & 0 & 0 & 1 & 0\\ 0 & 0 & 0 & 0 & 0 & 0 & 0 & 0 & 0 & 1 \end{array}\right]$ is the system transfer matrix; and $w_{t}^{k}$ and $v_{t}^{k}$ are the independent zero-mean Gaussian noise variables to process and measure with covariance $R^{w,k}$ and $R^{v,k}$, respectively.

#### 3.2.1. Predicting Dynamic Obstacles

The motion state and its covariance matrix of each dynamic obstacle in the present frame from the last frame is predicted as follows:

(11) $${\hat{S}}_{t+1}^{k}=AS_{t}^{k}$$

The covariance ${\hat{P}}_{t+1}^{k}$ for ${\hat{S}}_{t+1}^{k}$ is estimated as follows:

(12) $${\hat{P}}_{t+1}^{k}=AP_{t}^{k}A^{T}+R_{t}^{w,k}$$

#### 3.2.2. Updating Dynamic Obstacles on a Road

In this section, data association is performed between the newly detected obstacle point and the historical dynamic obstacle list. Once a point is designated as an obstacle by applying the rules in Section 2.1, the point is tested to determine whether it is located on the road. A road is static and the measurement of the sensors in the vehicle and is accurate; hence, the predicted road area between the predicted road curb obtained in Section 3.1.2 is employed. If the projection of the point onto the *x* − *y* plane is not on the road surface, then the point is not clustered because it does not affect driving decision. Otherwise, it is designated as a road obstacle. The point will be matched first with the dynamic obstacles in the historical dynamic obstacle list. If the match succeeds, then the motion state of the matched dynamic obstacle will be updated. Otherwise, a new dynamic obstacle will be created.

The neighboring pixels will be searched to determine whether they are attached to a cluster. If a neighboring pixel is attached, then it is included in the same cluster. Otherwise, the pixel is evaluated to identify the cluster to which it belongs to. For points P=(x,y) and cluster ${\text{ς}}_{t}^{k}$, the process is as follows:

(13) $$\vec{n_{t}^{k}}=(\cos({\text{ϑ}}_{t}^{k}),\sin({\text{ϑ}}_{t}^{k})$$

(14) $$d_{t}^{k,l}=|\vec{O_{t}^{k}P}*\vec{n_{t}^{k}}|$$

(15) $$d_{t}^{k,w}=||\vec{O_{t}^{k}P}\times \vec{n_{t}^{k}}||$$

(16) $$pos_{t}^{k}=\frac{1}{\sqrt{2\pi }L_{t}^{k}W_{t}^{k}}e^{-\frac{1}{2}(\frac{d_{t}^{k,l2}}{L_{t}^{k2}}+\frac{d_{t}^{k,w2}}{W_{t}^{k2}})}$$

The largest pos among ${pos}_{t}^{k}$ is determined. If a pos is less than the threshold, then the point is dealt with, as indicated in Section 2.2. Otherwise, the *k*th cluster is updated with the new point. The process is shown as follows:

(17) $$pos=\underset{k}{\max}(pos_{t}^{k})\{\begin{array}{c} \geq \delta_{p}\;\;\;\;\;\;\;\;\;update\;the\;\varsigma_{t}^{k}\\ <\delta_{p}\;\;\text{dealt}\;\text{as}\;\text{shown}\;\text{in}\;\text{Section}\;2.2 \end{array}$$

After processing all the points, a list of clusters is established for this frame. If a cluster is newly detected, then it is initialized as described earlier. Otherwise, the observed state vector $z_{t}^{k}={\left[x_{t}^{k}y_{t}^{k}v_{t}^{x,k}v_{t}^{y,k}a_{t}^{x,k}a_{t}^{y,k}{\text{ϑ}}_{t}^{k}\alpha_{t}^{k}L_{t}^{k}W_{t}^{k}\right]}^{T}$ can be obtained as follows.

$O_{t}^{k}=(x_{t}^{k},y_{t}^{k})$, ${\text{ϑ}}_{t}^{k}$, and $L_{t}^{k},W_{t}^{k}$ can be identified through the process described in Section 2.3.

The other components of the state vector can be obtained as follows:

(18) $$v_{t}^{x,k}=\frac{x_{t}^{k}-x_{t-1}^{k}}{T}$$

(19) $$v_{t}^{y,k}=\frac{y_{t}^{k}-y_{t-1}^{k}}{T}$$

(20) $$a_{t}^{x,k}=\frac{v_{t}^{x,k}-v_{t-1}^{x,k}}{T}$$

(21) $$a_{t}^{y,k}=\frac{v_{t}^{y,k}-v_{t-1}^{y,k}}{T}$$

(22) $$\alpha_{t}^{k}=\frac{{\text{ϑ}}_{t}^{k}-{\text{ϑ}}_{t-1}^{k}}{T}$$

The state vector and its covariance are updated as follows:

(23) $$Kg_{t}^{k}=\frac{{\hat{P}}_{t}^{k}}{{\hat{P}}_{t}^{k}+R_{v,k}}$$

(24) $$S_{t}^{k}={\hat{S}}_{t}^{k}+Kg_{t}^{k}(Z_{t}^{k}-{\hat{S}}_{t}^{k})$$

(25) $$P_{t}^{k}=(I-Kg_{t}^{k}){\hat{P}}_{t}^{k}$$

## 4. Results and Discussion

The experiment was performed on our UGV, called the *Intelligent Pioneer*, which was equipped with various sensors, e.g., a camera, an infrared camera, an Ibeo LIDAR, a Sick LIDAR, a Velodyne LIDAR, and SPAN-CPT. The sensors used in this work were the Velodyne LIDAR and SPAN-CPT. The processor used for the LIDAR data was a Core™ i7-3610QE with a process frequency that could be increased to 3.3 GHz and a cache of 6 M.

**Figure 7:**
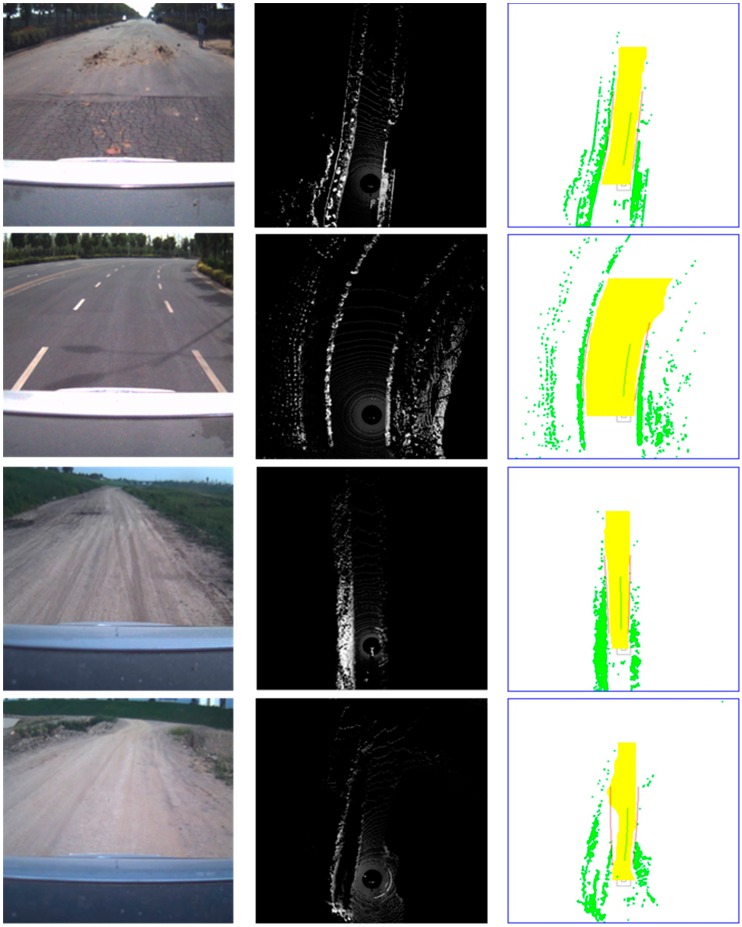
(**Left column**) Original image taken by a camera; (**Middle column**) Original grid map obtained by the Velodyne LIDAR; (**Right column**) Ground segmentation and curb fitting results.

First, ground segmentation experiments were conducted under various road conditions. The proposed multi-feature ground segmentation algorithm was proven to be effective under various road conditions. The experiment was performed in both urban and rural environments. Four representative scenes are shown in Figure 7.

The original image obtained using the camera and the original grid map developed by the Velodyne LIDAR are shown in the first and second columns, respectively, whereas the results of ground segmentation and curb fitting are shown in the third column. The yellow area denotes the drivable area, whereas the red and green lines represent the road curb and driving guide line, respectively.

**Figure 8:**
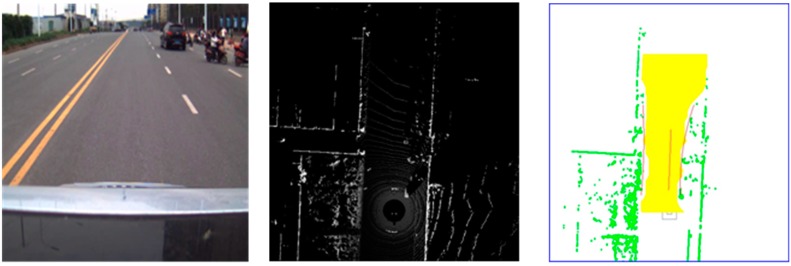
Scene where the vehicle parked along the road side was designated as the road curb. (**Left column**) Original image taken by camera; (**Middle column**) Original grid map obtained by Velodyne LIDAR; (**Right column**) Ground segmentation and curb fitting results.

Two urban scenes are shown in the first two rows. The first row shows a straight road that is not flat. On the one hand, the road surface is cracked. On the other hand, it is not clean because of the presence of mud. Given such disturbances, the points projected onto the road surface do not form a smooth circle but an arc with a high-frequency noise. However, based on the third column, the road surface was detected as clean and without noise. This result was achieved by adopting features in two aspects: the local point characteristics described in Section 2.1 and the local area continuity feature processed similarly in Section 2.4. Multi-feature road surface segmentation removed nearly all the noise points on the road surface because a multi-feature scheme with a relatively looser threshold was applied. The remaining noise points, if they existed, were eroded by the surrounding continuous road surface area. The curve shown in the second row demonstrates the capability of the algorithm to detect road curbs. In general, the curve fits the general road curb well. However, some gap exists in our experiment; these gaps are mainly caused by two reasons. First, the curve is a quadratic, and thus, it may be unsuitable for a curb in an actual scene. Second, the dynamic obstacle near a road curb will be detected as a road curb. However, driving decision will be unaffected because the continuity of the drivable area is not influenced by the dynamic area, as shown in Figure 8. As shown in this figure, the packed cars and pedestrians along a road curb are detected as the road edge, which causes the irregularity of the road curb. However, this phenomenon will not affect driving decision because the given drivable area is reasonable.

The third and fourth rows in Figure 7 depict the rural environment, wherein the boundary is represented by bushes, *i.e.*, positive obstacles, in the third row, whereas the boundary is represented by a ditch, *i.e.*, a negative obstacle, in the fourth row. The challenge in ground segmentation in a rural environment is considerably greater than that in an urban environment because points projected onto the road surface are irregular. Moreover, diffuse reflection occurs because road surface is coarse. Hence, the local point feature becomes increasingly irregular. In the experiment conducted in the rural environment, height measurements were inaccurate because of the position of the horizontal projection, which might be caused by the jolt of the vehicle in the rural environment. Based on the analysis, the local coordinate system keeps changing with the fluctuation in pitch of the vehicle during a jolt, which results in the incorrect calculation of the *z* value of the point. Each single feature was tested in our experiment, and the results were unsatisfactory. However, combining multiple features yielded an excellent result.

**Figure 9:**
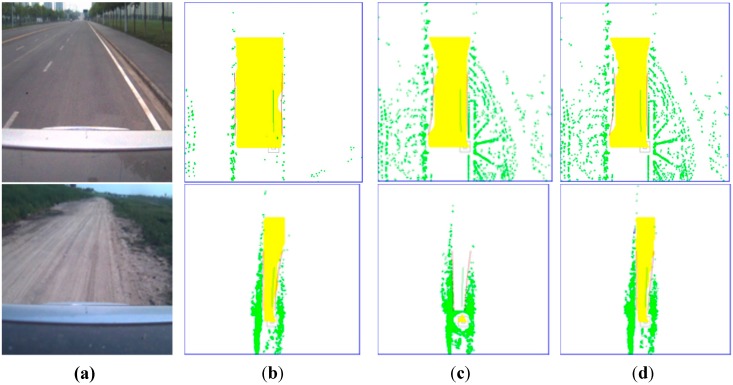
Comparison between multi-feature road surface segmentation and segmentation based on height difference. Left to right columns: (**a**) Original image taken by a camera; (**b**) Image resulting from the height difference feature with a strict threshold; (**c**) Image resulting from the height difference feature with a loose threshold; (**d**) Image resulting from the multi-feature scheme with a loose threshold.

The vehicle drives under various road conditions; hence, the LIDAR finds performing effective ground segmentation to be difficult because of the irregularity of the source point cloud, which results from vehicle fluctuation and the diffused reflection of the ground. To address this problem, the philosophy behind ground segmentation is a multi-feature scheme with a relatively looser threshold. Experiments were conducted by applying different algorithms under various road surface conditions in ground segmentation to exhibit the advantages of the multi-feature scheme. Height difference is a widely applied characteristic in ground segmentation; hence, a comparison between segmentation that applies height difference and segmentation that applies the fusion of features is presented in Figure 9. The two scenes shown in the figure represent urban and rural environments. This figure indicates that when road surface is flat and road curb is relatively low, a loose threshold is more suitable when applying the single height difference feature because the strict threshold classifies a true road curb as road surface. The trees on the roadside are classified as road curbs instead, which is shown in the second column of the first row. However, the environment is more complex in rural areas. Based on the image, nearby points are classified as non-ground points and road surface is restricted to a small area.

**Figure 10:**
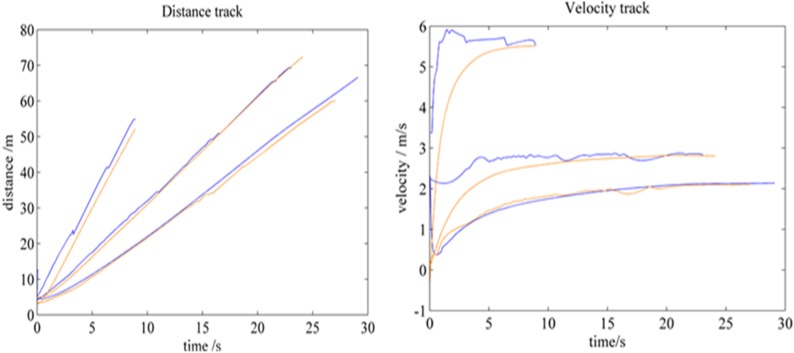
Tracking experiment on the distance and velocity of a vehicle under various velocities.

In tracking dynamic obstacles; an experiment was performed to track vehicles under various velocities. The positions and velocities of a target vehicle that was moving at various velocities were recorded by CPT. Meanwhile; the test vehicle was stopped to estimate the position and velocity of the target vehicle. Three groups of experiments are shown in Figure 10; with speeds of approximately 2; 3; and 6 m/s. The data obtained by CPT are shown in blue; whereas the estimations are indicated in saffron yellow. The top image illustrates the track of the distance of the target vehicle from the test vehicle; whereas the bottom image shows the track of the velocity of the target vehicle. This figure also indicates that tracking under a low speed is accurate; whereas tracking under a high speed is relatively inaccurate. Based on the analysis; this finding is caused by two reasons. First; the result occurred because of the property of the Kalman filter; which considers previous information. Thus when relative speed is high; the frames that can be tracked for the target vehicle are few. Second; this phenomenon occurred because fluctuation increases with speed. However; the relative speed between the test vehicle and the target vehicle is low. Thus; when the vehicle is on an actual driving environment; our algorithm exhibits good capability in tracking the vehicle.

**Figure 11:**
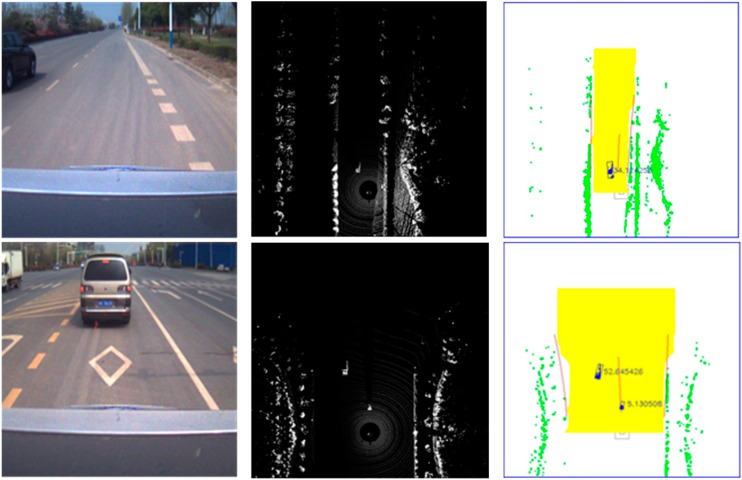
Dynamic obstacle track result. (**Left column**) Original image taken by a camera; (**Middle column**) Original grid map obtained by the Velodyne LIDAR; (**Right column**) Ground segmentation and dynamic obstacle track result.

The two scenes in Figure 11 show our experiment conducted in an actual driving environment. The detected dynamic obstacle is marked in blue and surrounded by a minimum bounding box. The number on the right column denotes the velocity of the target vehicles. As shown in Figure 11, the first row indicates the track of a single vehicle on a straight road, where the test vehicle is stopped by the road curb. As the test vehicle stops, the velocity of the target vehicle is reflected. However, this velocity is the relative velocity between the test vehicle and the target vehicle. Hence, the vehicle moving toward the opposite direction has high speed, whereas the velocity of the vehicle moving in the same direction is relatively low. This phenomenon is illustrated in the second scene, which represents a crossroad environment. The left target vehicle is moving toward the opposite direction with higher velocity, whereas the right vehicle is moving toward the same direction with lower velocity.

**Figure 12:**
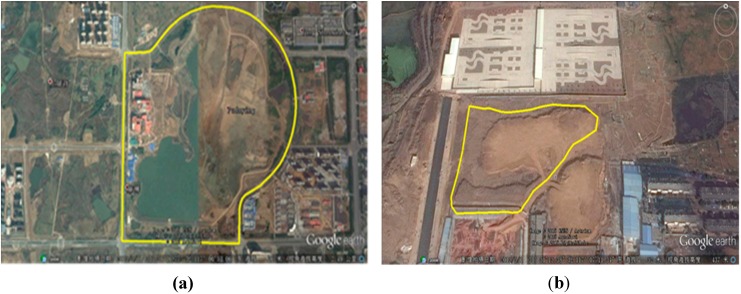
Satellite aerial photographs of the urban scene (**a**) and the rural scene (**b**).

To evaluate the real performance of the proposed algorithm, experiments on urban and rural environments were conducted. The satellite aerial photographs of two scenes are shown in Figure 12. The urban scene Figure 12a is composed of various road conditions: straight roads, crossroads, and curved roads. In the rural environment Figure 12b, the road condition is more complex. The bad road condition causes the vehicle to jolt, which makes the generated point cloud more irregular. The length of the route is about approximately 5 km in Figure 12a and approximately 800 m in Figure 12b.

**Figure 13:**
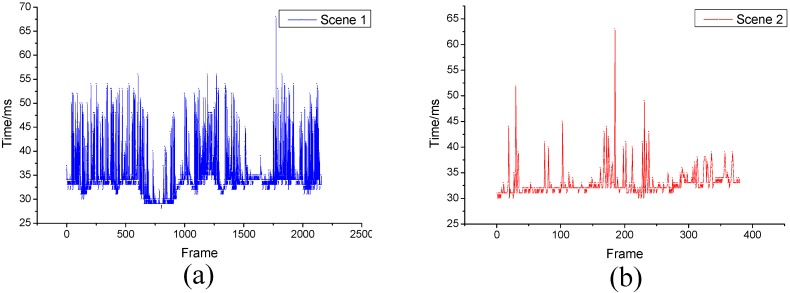
Real-time performances of the urban scene (**a**) and the rural scene (**b**).

First, the real-time performance for each frame is obtained and the result is shown in Figure 13. As shown in the figure, the real-time performances of the urban scene and the rural scene are nearly the same. Moreover, the figure shows that the real-time performance is invariant to time.

**Figure 14:**
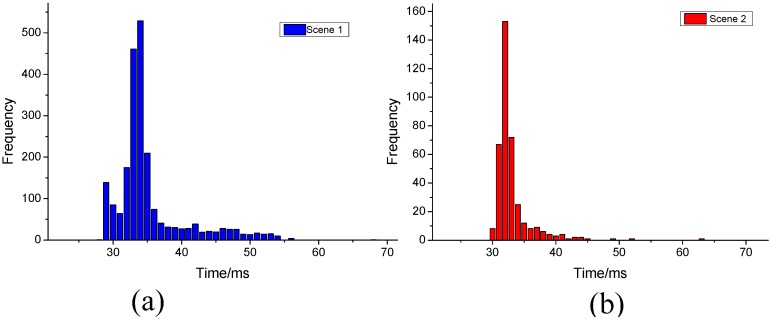
Distribution of process time for each frame in the urban scene (**a**) and the rural scene (**b**).

The distribution of process time for each frame is illustrated in Figure 14. As shown in the figure, most of the samples fall within [30,40] in both cases.

An experiment on the robustness of road curb detection was also conducted. Ground segmentation is the key procedure for an effective of the road curb detection. As shown in our experiment, accurate ground segmentation can guarantee the effectiveness of road curb detection and a minor segmentation error was tolerable because of the error elimination process in the following step. Only significant errors will lead to incorrect detection of the road curb. Thus, a statistic is proposed. This statistic consists of four contents: the percentage of successful ground segmentation, the percentage of ground segmentation with minor errors whereas wherein the road curb detection is not affected, the percentage of incorrect ground segmentation which caused leak detection of the road, and the percentage of incorrect ground segmentation which caused false detection of the road. The results are shown in Table 2. As indicated in the table, performance is better in urban scenes, wherein road conditions are better than in rural scenes. Leak detection of the road curb occurred in cases wherein the road curb is not distinctive from the road surface. However, false detection occurred in crossroad situations which are not tackled in this work in urban scenes and in those cases wherein the road situation is severe in rural scenes.

**Table 2:** Statistics on ground segmentation and road curb detection.

Scene	Accurate	Minor Error	Leak Detection	False Detection
Scene 1	97.44%	1.85%	0.55%	0.16%
Scene 2	89.29%	6.54%	1.56%	2.61%

## 5. Conclusions/Outlook

A framework for the application of a multi-line LIDAR to model key elements that comprise the driving environment, *i.e.*, road curbs and dynamic obstacles, is proposed in this work. The framework combine the modeling of the road information and the road dynamic obstacle as an organic entirety. This approach is proven to be robust and satisfied the requirement for the online process in the experiment presented in earlier sections. A quick and robust modeling of dynamic obstacles and road curbs consists of several procedures that all pose a significant influence on the final result. To segment the ground robustly, in contrast to the application of single or very few features in ground segmentation in previous work, the philosophy of multi-features with loose thresholds is employed in this work, which proves to be adaptive to various environments. As for the dynamic obstacles, in contrast to previous works where dynamic obstacles are clustered after the grid map is generated, dynamic obstacles are clustered in this work once they are classified as non-ground points. The latter approach has two benefits. First, the different strategies are applied depend on the neighborhood information of the tested point. When the neighbor points of the test point are designated as non-ground points, the possibility that the test point is a non-ground point increases, and thus, a looser threshold can be applied for the point to be designated as non-ground point. Second, clustering time is reduced because clustering is performed locally instead of globally. Moreover, shape features are calculated by adopting the Karhunen-Loeve transformation, which is the first time such a procedure is employed. In the tracking process, the shape features mentioned before are used to fuse current information with historical information. In road curb detection, the distance image is applied to detect the road curb, whereas the snake model is used for tracking because of its capability to consider both local and global information. To our knowledge, the snake model has never been applied in road curb tracking for LIDAR data processing. By modeling road area, a vehicle can determine the path of its movement. By modeling dynamic obstacles on the road, a vehicle can determine places to avoid. The presented framework aimed to provide a novel framework for the application of multi-beam LIDAR under various road environment. Unlike the frameworks presented before, the purpose of the presented framework is to dig deep into the process of the application of multi-beam LIDAR to find a better way to organize the information flow. The robustness and the real-time performance are contradictory to some extent. The way to solve this problem is by finding a way to combine processes as much as possible. By combining different process together, information loss is eliminated between different procedures. What’s more, the process time is reduces because the application of local vicinity information. The framework is tested under various road conditions and proved to be able to model the road curb and the dynamic road obstacle robustly on-line. Further research will focus on simultaneous location and mapping using multi-beam LIDAR by integrating local and global information.

## References

[1] Dollar P., Wojek C., Schiele B., Perona P. (2012). Pedestrian Detection: An Evaluation of the State of the Art. IEEE Trans. Pattern Anal. Mach. Intell..

[2] Andriluka M., Roth S., Schiele B. People-tracking-by-detection and people-detection-by-tracking. Proceedings of the 2008 IEEE Conference on Computer Vision and Pattern Recognition (CVPR).

[3] Yinghua H., Hong W., Bo Z. (2004). Color-based road detection in urban traffic scenes. IEEE Trans. Intell. Transp. Syst..

[4] Zhao J., Katupitiya J., Ward J. Global correlation based ground plane estimation using V-disparity image. Proceedings of the 2007 IEEE International Conference on Robotics and Automation.

[5] Sivaraman S., Trivedi M.M. (2010). A general active-learning framework for on-road vehicle recognition and tracking. IEEE Trans. Intell. Transp. Syst..

[6] Arrospide J., Salgado L., Marinas J. HOG-like gradient-based descriptor for visual vehicle detection. Proceedings of the 2012 IEEE Intelligent Vehicles Symposium (IV).

[7] Sun Z.H., Bebis G., Miller R. (2006). On-road vehicle detection: A review. IEEE Trans. Pattern Anal. Mach. Intell..

[8] Junjie H., Huawei L., Zhilin W., Tao M., Yan S. Robust lane marking detection under different road conditions. Proceedings of the 2013 IEEE International Conference on Robotics and Biomimetics (ROBIO).

[9] Kelly P., O’Connor N.E., Smeaton A.F. (2008). A framework for evaluating stereo-based pedestrian detection techniques. IEEE Trans. Circuits Syst. Video Technol..

[10] Soga M., Kato T., Ohta M., Ninomiya Y. Pedestrian detection with stereo vision. Proceedings of the 2005 21st International Conference on Data Engineering Workshops.

[11] Giosan I., Nedevschi S., Bota S. Real time stereo vision based pedestrian detection using full body contours. Proceedings of the IEEE 5th International Conference on Intelligent Computer Communication and Processing (ICCP 2009).

[12] Lin Y.C., Chan Y.M., Chuang L.C., Fu L.C., Huang S.S., Hsiao P.Y., Luo M.F. Near-Infrared Based Nighttime Pedestrian Detection by Combining Multiple Features. Proceedings of the 2011 14th International Ieee Conference on Intelligent Transportation Systems (ITSC).

[13] Cao Y.Y., Pranata S., Nishimura H. Local Binary Pattern Features for Pedestrian Detection at Night/Dark Environment. Proceedings of the 2011 18th Ieee International Conference on Image Processing.

[14] Brehar R., Vancea C., Nedevschi S. Pedestrian Detection in Infrared Images Using Aggregated Channel Features. Proceedings of the 2014 IEEE International Conference on Intelligent Computer Communication and Processing (ICCP).

[15] Ge J.F., Luo Y.P., Tei G.M. (2009). Real-Time Pedestrian Detection and Tracking at Nighttime for Driver-Assistance Systems. IEEE Trans. Intell. Transp. Syst..

[16] O'Malley R., Jones E., Glavin M. (2010). Detection of pedestrians in far-infrared automotive night vision using region-growing and clothing distortion compensation. Infrared Phys. Technol..

[17] Fardi B., Weigel H., Wanielik G., Takagi K. Road border recognition using fir images and lidar signal processing. Proceedings of the Intelligent Vehicles Symposium.

[18] Shin Y., Jung C., Chung W. Drivable Road Region Detection Using a Single Laser Range Finder for Outdoor Patrol Robots. Proceedings of the 2010 IEEE Intelligent Vehicles Symposium (IV).

[19] Wijesoma W.S., Kodagoda K.R.S., Balasuriya A.P. (2004). Road-boundary detection and tracking using ladar sensing. IEEE Trans. Robotics. Autom..

[20] Jaehyun H., Dongchul K., Minchae L., Myoungho S. (2012). Enhanced road boundary and obstacle detection using a downward-looking LIDAR sensor. IEEE Trans. Veh. Technol..

[21] Kodagoda K.R.S., Ge S.S., Wijesoma W.S., Balasuriya A.P. (2007). IMMPDAF approach for road-boundary tracking. IEEE Trans. Veh. Technol..

[22] Liu Z., Wang J.L., Liu D.X. (2013). A New Curb Detection Method for Unmanned Ground Vehicles Using 2D Sequential Laser Data. Sensors.

[23] Yao W., Hinz S., Stilla U. (2010). Automatic vehicle extraction from airborne LiDAR data of urban areas aided by geodesic morphology. Pattern Recognit. Lett..

[24] Yao W., Hinz S., Stilla U. (2011). Extraction and motion estimation of vehicles in single-pass airborne LiDAR data towards urban traffic analysis. ISPRS J. Photogramm. Remote Sens..

[25] Borcs A., Benedek C. (2015). Extraction of Vehicle Groups in Airborne Lidar Point Clouds With Two-Level Point Processes. IEEE Trans. Geosci. Remote Sens..

[26] Zhang K.Q., Chen S.C., Whitman D., Shyu M.L., Yan J.H., Zhang C.C. (2003). A progressive morphological filter for removing nonground measurements from airborne LIDAR data. IEEE Trans. Geosci. Remote Sens..

[27] Douillard B., Underwood J., Kuntz N., Vlaskine V., Quadros A., Morton P., Frenkel A. On the segmentation of 3D LIDAR point clouds. Proceedings of the IEEE International Conference on Robotics and Automation.

[28] Pascoal R., Santos V., Premebida C., Nunes U. (2015). Simultaneous Segmentation and Superquadrics Fitting in Laser-Range Data. IEEE Trans. Veh. Technol..

[29] Vaskevicius N., Birk A., Pathak K., Schwertfeger S. (2010). Efficient Representation in Three-Dimensional Environment Modeling for Planetary Robotic Exploration. Adv. Robot..

[30] Petrovskaya A., Thrun S. (2009). Model based vehicle detection and tracking for autonomous urban driving. Auton. Robot..

[31] Azim A., Aycard O. Detection, Classification and Tracking of Moving Objects in a 3D Environment. Proceedings of the 2012 IEEE Intelligent Vehicles Symposium (IV).

[32] Chen T.T., Dai B., Wang R.L., Liu D.X. (2014). Gaussian-Process-Based Real-Time Ground Segmentation for Autonomous Land Vehicles. J. Intell. Robot. Syst..

[33] von Hundelshausen F., Himmelsbach M., Hecker F., Mueller A., Wuensche H.-J. (2008). Driving with tentacles: Integral structures for sensing and motion. J. Field Robot..

[34] Urmson C., Anhalt J., Bagnell D., Baker C., Bittner R., Clark M.N., Dolan J., Duggins D., Galatali T., Geyer C. (2008). Autonomous driving in urban environments: Boss and the Urban Challenge. J. Field Robot..

[35] Kammel S., Ziegler J., Pitzer B., Werling M., Gindele T., Jagzent D., Schroder J., Thuy M., Goebl M., von Hundelshausen F. (2008). Team AnnieWAY’s autonomous system for the 2007 DARPA Urban Challenge. J. Field Robot..

[36] Moosmann F., Pink O., Stiller C. Segmentation of 3D lidar data in non-flat urban environments using a local convexity criterion. Proceedings of the 2009 IEEE Intelligent Vehicles Symposium.

[37] Montemerlo M., Becker J., Bhat S., Dahlkamp H., Dolgov D., Ettinger S., Haehnel D., Hilden T., Hoffmann G., Huhnke B. (2008). Junior: The Stanford entry in the Urban Challenge. J. Field Robot..

[38] Thrun S., Montemerlo M., Dahlkamp H., Stavens D., Aron A., Diebel J., Fong P., Gale J., Halpenny M., Hoffmann G. (2006). Stanley: The robot that won the DARPA Grand Challenge. J. Field Robot..

[39] Bohren J., Foote T., Keller J., Kushleyev A., Lee D., Stewart A., Vernaza P., Derenick J., Spletzer J., Satterfield B. (2008). Little Ben: The Ben Franklin Racing Team’s entry in the 2007 DARPA Urban Challenge. J. Field Robot..

[40] Zhao G., Yuan J. Curb detection and tracking using 3D-LIDAR scanner. Proceedings of the 2012 19th IEEE International Conference on Image Processing (ICIP).

[41] Simon D. (2010). Kalman filtering with state constraints: A survey of linear and nonlinear algorithms. IET Contr. Theory Appl..

[42] Jian L., Huawei L., Zhiling W. A framework for detecting road curb on-line under various road conditions. Proceedings of the 2014 IEEE International Conference on Robotics and Biomimetics (ROBIO).

[43] Kass M., Witkin A., Terzopoulos D. (1987). Snakes–Active contour models. Int. J. Comput. Vis..

[44] Williams D.J., Shah M. (1992). A fast algorithm for active contours and curvature estimation. Cvgip Image Underst..

